# Electron polarons in the subsurface layer of Mo/W-doped BiVO_4_ surfaces†

**DOI:** 10.1039/c8ra09009b

**Published:** 2019-01-08

**Authors:** Jianhang Cen, Shunning Li, Jiaxin Zheng, Feng Pan

**Affiliations:** School of Advanced Materials, Peking University, Shenzhen Graduate School Shenzhen 518055 People's Republic of China lisn@pkusz.edu.cn panfeng@pkusz.edu.cn

## Abstract

Monoclinic BiVO_4_ has been regarded as a promising photocatalyst for water splitting in recent years. In this research, the effects of Mo/W dopants near the surfaces of BiVO_4_ on electron transport are investigated using first-principles calculations. We demonstrate that the additional electron introduced by Mo/W either in the bulk or near the surfaces forms a self-trapped small polaron. The polaron prefers to be localized on the transition metal ions in the subsurface layer when Mo/W is doped in the vicinity of the surfaces. The localized positions of polarons can be rationalized by the d-orbital energy levels of the transition metals and the variation of electrostatic potential. The concentrated electron polarons in the subsurface layer of BiVO_4_ surfaces can build fast lanes for electron migration and mitigate the electron–hole recombination process, which underlines the importance of dopants near the surfaces as compared with those in the bulk.

## Abstract 2

Monoclinic BiVO_4_ has been regarded as a promising photocatalyst for water splitting in recent years.

## Graphical abstract

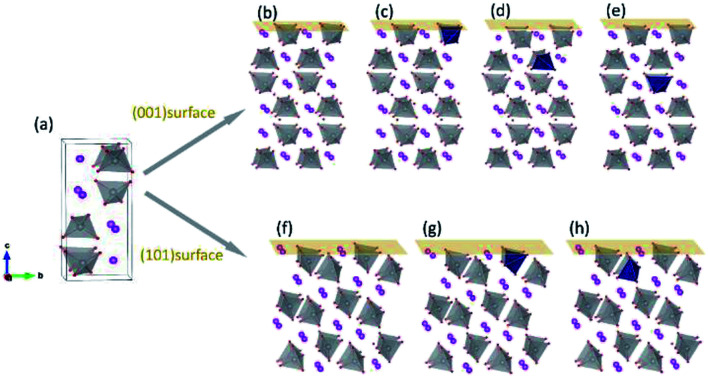

## Introduction

Due to the continuous depletion of fossil fuel resources and the environmental concerns associated with their combustion, technologies for solar energy conversion, such as photoelectric devices and photocatalytic water splitting, have dramatically grown in importance in the past decades. Although the first experiment for photocatalytic water splitting dates back to the 1970s,^1^ it is not entirely surprising that this solar-to-chemical pathway is still far from practical application, owing to the lack of successful electrocatalysts that meet the requirements of high activity, long durability and low costs.^2^ This technology comprises two main electrochemical processes: the hydrogen evolution reaction (HER)^3^ and oxygen evolution reaction (OER)^4^ at the cathode and the anode of an electrolytic cell, respectively. The OER process has long been considered as the bottleneck that dictates the over-potential required to reach an operating current density for photocatalytic water splitting. Therefore, considerable efforts have focused on the exploration of electrocatalysts^5,6^ that not only absorb in the visible-light region, but also have suitable band edge alignment with respect to water redox potentials so that they can effectively drive the OER. Previous studies have yielded several promising candidates, including BiVO_4_, Fe_2_O_3_ and WO_3_.^7^ Among these oxide semiconductors, BiVO_4_ stands out due to its narrow bandgap (2.4–2.5 eV),^8–10^ high stability towards anodic photocorrosion and good producibility by cost-effective methods.^5,10–13^ Additionally, BiVO_4_ possesses the advantages of non-toxicity and abundance in nature.^14^ Nevertheless, the intrinsically poor electron conductivity and the resultant significant electron–hole recombination^15,16^ tend to undermine its practical use in large-scale application, which necessitates further modification to increase either the concentration or the mobility of electrons in BiVO_4_.

As widely reported, the intrinsic properties of semiconductors or insulators can be permanently altered by the introduction of intentional dopants in the bulk or near the surfaces.^17,18^ Therefore, it is highly desirable to design suitable n-type dopants in BiVO_4_ so as to achieve sufficient electron conductivity for promoting the photocatalytic performance. Mo and W, both containing one more valence electron as compared with V, have been proved to be the ideal candidates in recent experiments.^14,19–24^ It was proposed that Mo dopants can increase the carrier density in BiVO_4_ and possibly enlarge the hole diffusion length.^21,23^ Similar claims were made for W-doped BiVO_4_ with carrier density estimated to be twice that of pure BiVO_4_.^19,24^ What is interesting is that the superficial amount of Mo and W dopants were found to be more involved in the improvement of electrochemical performance than those in the bulk.^25^ This result implies that Mo/W-doped BiVO_4_ surfaces may have distinct roles in increasing the concentration or mobility of electrons. Another issue also worth mentioning is that the photo-generated electrons, in the case of pristine BiVO_4_, were theoretically predicted to be trapped as polarons on V ions.^26^ Therefore, a comprehensive understanding of polaronic charge carriers generated by Mo/W dopants near the surfaces is timely and significant, which may shed light on the origins of the improved OER activity observed in the previous experiments.

In this work, we perform a detailed first-principles analysis of the effects of Mo and W dopants near the surfaces of monoclinic BiVO_4_. The (001) and (101) surfaces are selected as representatives, since many researchers have referred to them as the most stable surfaces in the equilibrium morphology of BiVO_4_.^8,27–29^ Our results show that small polarons can be generated by Mo and W dopants in the vicinity of (001) and (101) surfaces. The polarons would be preferentially localized on V ions in the subsurface layer, thus retarding the electron–hole recombination process at the surface layer where the photo-generated holes are to take part in the OER. The fast lanes for electron transport in the subsurface layer may also add to the promotion of photocatalytic performance of BiVO_4_. These results underline the importance of dopants near the surfaces as compared with those in the bulk.

## Computational methodology

All calculations were performed by using the density functional theory (DFT), implemented in the Vienna *Ab Initio* Simulation Package (VASP).^30^ The generalized gradient approximation (GGA) of Perdew–Burke–Ernzerhof (PBE) form was used for the exchange-correlation functional.^31^ In order to reduce the self-interaction errors, we chose the DFT+*U* scheme as introduced by Dudarev *et al.*^32^ Effective *U* values of 3.2, 2.3 and 2.1 eV for V, Mo and W, respectively, were taken from literature.^32^ The valence configurations were treated as 6s^2^6p^3^ for Bi, 3d^3^4s^2^ for V, 2s^2^2p^4^ for O, 4d^4^5s^2^ for Mo and 5d^4^6s^2^ for W. The cutoff energy for plane-wave basis functions was 520 eV. For the *k*-space sampling, 3 × 3 × 3 and 3 × 3 × 1 Gamma centered grids were employed in the calculations of supercells and surfaces, respectively. We have verified that the *k*-point grids were able to guarantee the accuracy of the results. Spin polarization was taken into account in all the calculations.

**Fig. 1:**
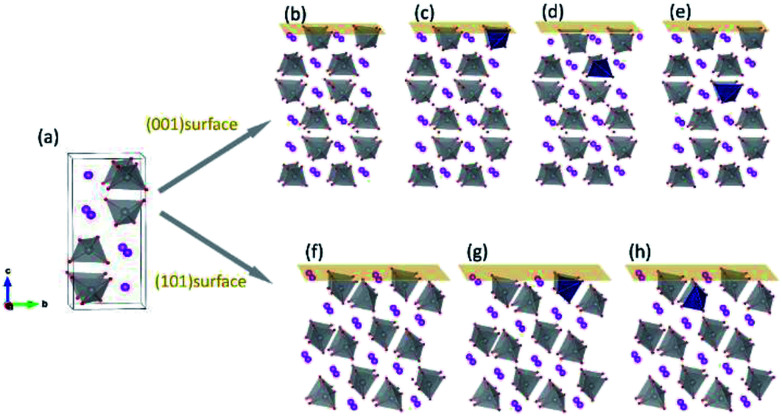
Crystal structure of monoclinic BiVO_4_. (a) Unit cell of BiVO4; (b) pristine (001) surface and the surfaces with dopants in the (c) first, (d) second and (e) third layers; (f) pristine (101) surface and the surfaces with dopants in the (g) first and (h) second layers. Red: O; gray: V; pink: Bi; dark blue: dopant atoms.

Fig. 1(a) shows the crystal structure of monoclinic BiVO_4_. Every V atom is surrounded by four O atoms, forming a distorted VO_4_ tetrahedron. After atomic relaxation, we derived the lattice parameters of monoclinic-BiVO_4_ structure (space group *I*2/*b*): *a* = 5.21 Å, *b* = 5.16 Å, *c* = 11.74 Å, and *γ* = 90.21°, which are close to the experiment values: *a* = 5.19 Å, *b* = 5.09 Å, *c* = 11.70 Å, and *γ* = 90.4°.^33^ The Mo/W atom was introduced into the 2 × 2 × 1 supercell (containing 16 bismuth atoms, 16 vanadium atoms and 64 oxygen atoms).

For surface relaxation, we made sure that the top and bottom surface are the same and relaxed them simultaneously to avoid the introduction of dipole moment along the *c* direction of the slabs. The thicknesses of the slabs for (001) and (101) surfaces are 16.50 Å and 13.08 Å respectively. A vacuum layer of 18 Å along *c* axis is thick enough to avoid the mirror interactions. The dopant atoms were positioned at different V sites in the vicinity of the surfaces, as shown in Fig. 1(b)–(h).

## Results

**Fig. 2:**
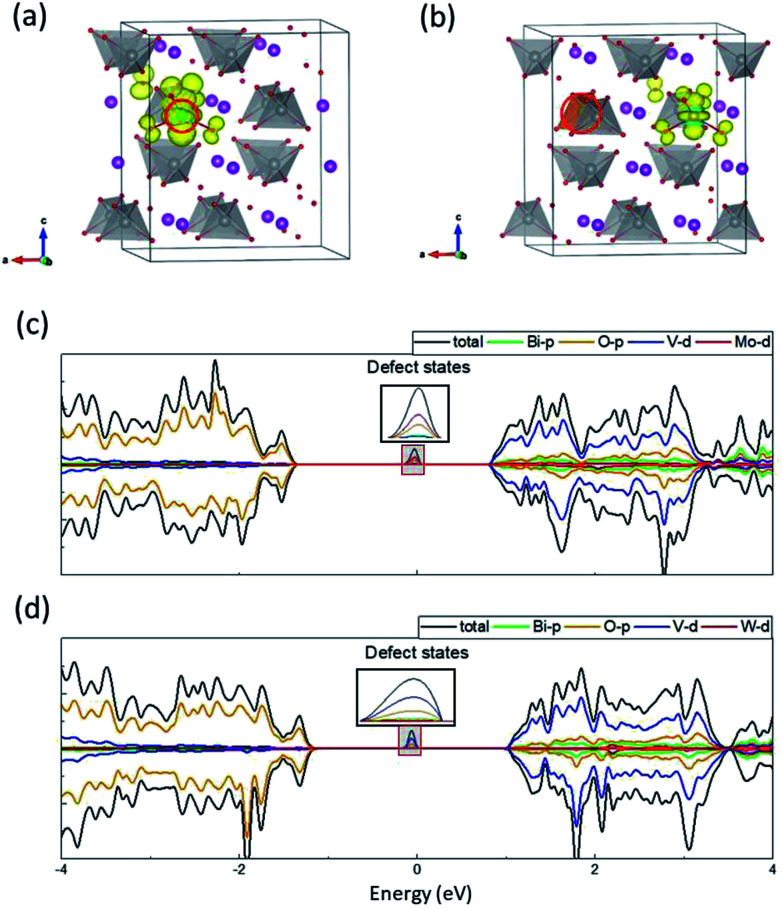
Electronic structure of Mo- and W-doped BiVO_4_. Charge density of defect states in (a) Mo- and (b) W-doped BiVO_4_. DOS for (c) Mo- and (d) W-doped BiVO_4_. The dopants are denoted by red circles.

To achieve a fundamental understanding of the effects of Mo and W dopants near BiVO_4_ surfaces, we first investigated their influence in the bulk. It should be mentioned that the valence band of BiVO_4_ is primarily composed of O-2p states, while the conduction band is characteristic of V-3d states with a little contribution from O-2p states (Fig. S1 in the ESI†), which is consistent with previous studies.^10,11^ The calculated band gap is around 2.34 eV, in good agreement with the experimental value (2.41 eV),^33^ which demonstrates the accuracy of the DFT+*U* scheme with the selected *U* values in our work. As shown in Fig. 2(a) and (b), one of the V atoms is replaced by Mo/W dopant, which induces a slight outward movement (∼0.05 Å) of each nearest-neighbor O ion around the dopant atom, as a result of the larger ionic radii of Mo (1.74 Å) and W (1.72 Å) than V (1.68 Å).^34^ We calculated the density of states (DOS) for the doped supercells, as depicted in Fig. 2(c) and (d). It is found that there exists a localized state in the band gap in both cases, which can be recognized as defect states lying around 1 eV beneath the conduction band minimum (CBM). What differs in both cases is that for Mo-doped BiVO_4_ the defect states are mainly made up of Mo-4d orbitals, while for W-doped BiVO_4_ the V-3d orbitals become populated.

Fig. 2(a) and (b) illustrate the charge density of the defect states for Mo- and W-doped BiVO_4_. The additional electron introduced by Mo is mainly localized on MoO_4_ while that introduced by W is on VO_4_. Both cases are characteristic of the formation of small polaron on the respective transition metal ion. A magnetic moment of ∼0.9 μ_B_ on the metal ion can further corroborate our claim. When we considered a delocalized electron in the supercell where all transition metal ions share the electron equally, the configuration would simultaneously transform into a polaronic state after structural relaxation. This implies that the delocalized state is unstable with respect to polaronic state. As such, the self-trapped energy, defined as the energy difference between the delocalized and polaronic states, can be calculated as the energy difference between CBM and the defect level, *i.e.* 0.91 and 1.13 eV for Mo and W dopants, respectively. These values are comparable to that in WO_3_ (∼1 eV), a typical semiconductor in which polaron hopping occurs.^35^ Our results coincide with previous research on the formation of electron polarons in BiVO_4_,^26^ and therefore we believe that the polaronic charge carriers generated by Mo/W dopants can exert significant influence on the catalytic performance of BiVO_4_ electrodes.

It should be mentioned that the difference in spatial charge distribution between Mo- and W-doped BiVO_4_ is originated from the d orbital energy levels of V, Mo and W. Previous studies have found that Mo-4d orbitals are slightly lower than V-3d, while W-5d orbitals have relatively higher energy level than V-3d.^12^ Consequently, the excess electron introduced by Mo dopant would prefer to fill up its own 4d orbitals, whereas the electron introduced by W would transfer to the 3d orbitals of its nearest-neighbor V ion. Nevertheless, the energy levels can be altered in the vicinity of surfaces, which might favor specific localized positions for the polarons introduced by dopants.

**Fig. 3:**
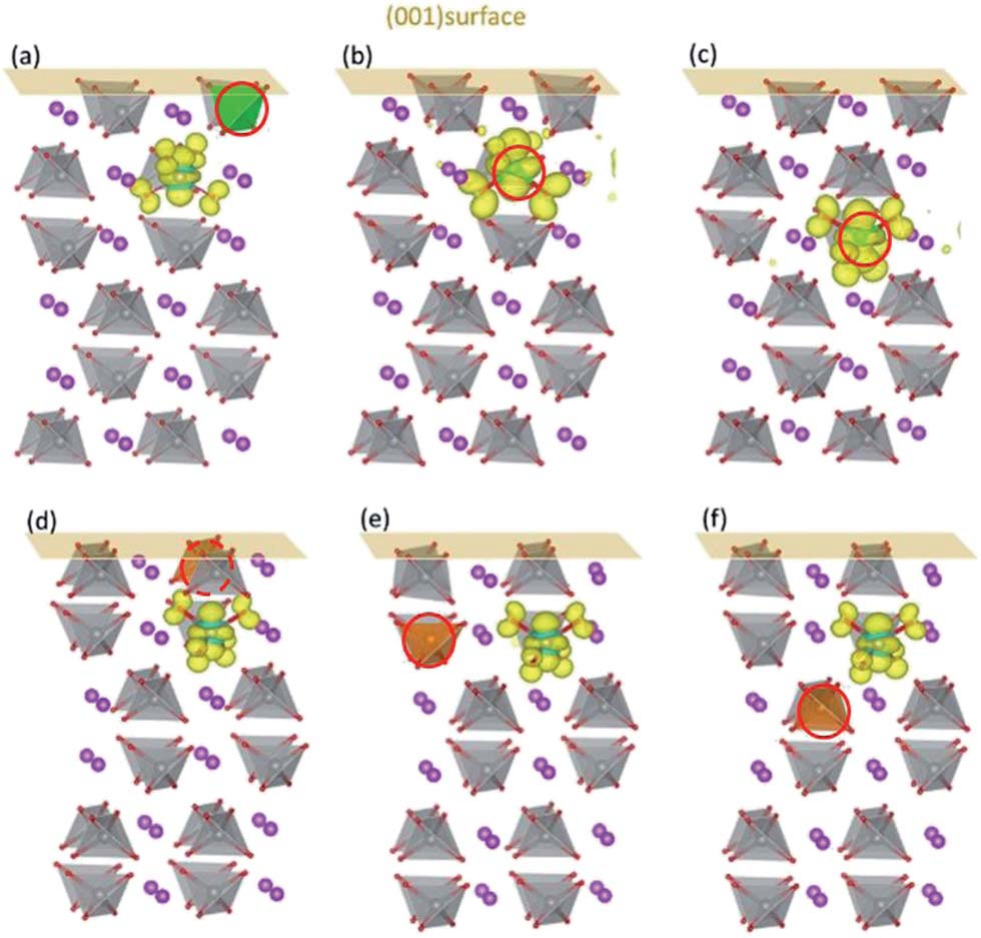
The charge density of defect states for Mo/W-doped (001) surfaces. Mo doped in (a) the first, (b) second and (c) third layers; W doped in (d) the first, (e) second and (f) third layers. The dopants are denoted by red circles.

Fig. 3 shows the influence of surfaces on the localized positions of polarons introduced by Mo/W dopants. When a V ion in the first layer of (001) surface is replaced by the dopant, the additional electron would be self-trapped in the same way for both cases (Fig. 3(a) and (d)), *i.e.*, forming small polaron on the V ion in the subsurface layer. These results are further substantiated by the DOS plotted in Fig. S2(a) and (c),† which indicates that the defect states are comprised of 3d orbitals of the corresponding V ion and 2p orbitals of the nearby O ions. When it comes to the second and third layer, however, the configuration differs between Mo and W dopants. Polarons introduced by Mo in the second or third layer would be localized on Mo ion, with DOS (Fig. S2(b) and (d)†) bearing strong resemblance to those in the bulk. Similarly, polarons generated by W in the second or third layer behave in the same pattern as that in the bulk, *i.e.*, localized on a V ion nearby.

**Fig. 4:**
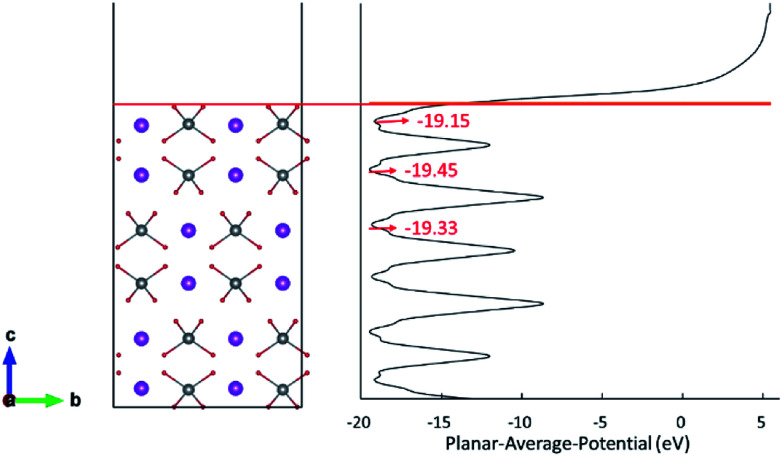
Planar-averaged electrostatic potential of the BiVO_4_ (001) surface.

The d orbital energy levels of V, Mo and W cannot rationalize the unexpected localized positions of polarons observed with dopants in the first layer (the surface layer). Therefore, we offer here another perspective in which electrostatic potential is viewed as one of the determinants. As shown in Fig. 4, the electrostatic potential is averaged in *ab* plane for BiVO_4_ (001) surface so as to capture the trend in the *c* direction. It is found that ions in the subsurface layer can produce lower averaged electrostatic potential than both the first and third layers, especially the first layer. We can anticipate that it would be more exothermic for electrons to localize on ions in the subsurface layer; in other words, the small polaron prefers to migrate from the dopant in the surface layer to V sites in the subsurface layer. Due to a relatively smaller difference in electrostatic potential between the second and third layers, the polaron would remain localized on Mo ion when it is doped in the third layer (Fig. 3(c)). Knowing that W would inevitably donate the excess electron, we are not surprised to see that polarons are all in the subsurface layer whenever W is doped in the first three layers (Fig. 3(d)–(f)). The influence of electrostatic potential can also be reflected by the local DOS for V atoms in these layers, as depicted in Fig. S3.† It is found that the CBM for (001) surface is dominated by V-3d orbitals in the subsurface layer, meaning that it would be energetically favorable for the photo-generated electrons in the conduction band to reside in that layer. As a consequence, electron polarons will be concentrated in the subsurface layer, which may contribute to a higher conductivity for electron transport.

**Fig. 5:**
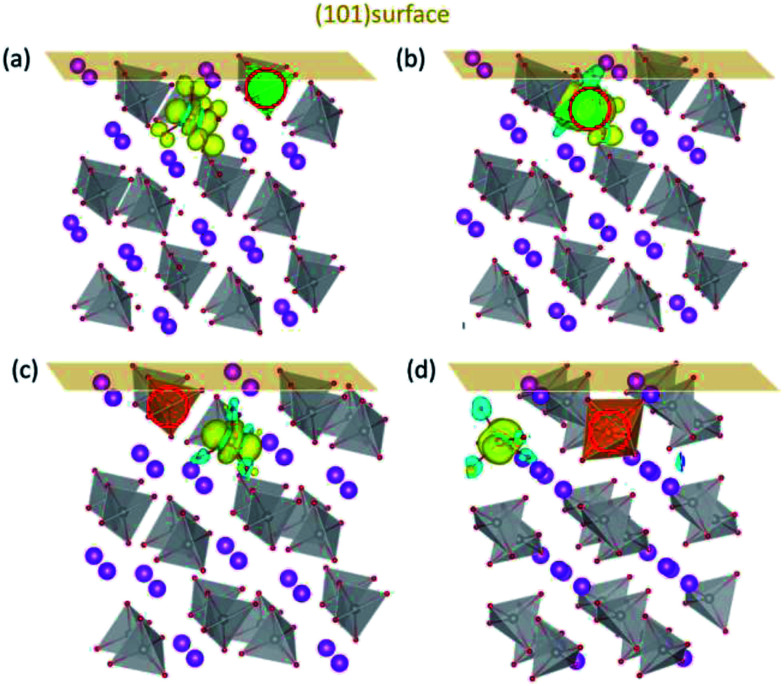
The charge density of defect states for Mo/W-doped (101) surfaces. Mo doped in (a) the first and (b) second layers; W doped in (c) the first and (d) second layers. The dopants are denoted by red circles.

The above features are retained for BiVO_4_ (101) surface. The DOS for (101) surfaces doped with Mo/W (Fig. S4†) are similar to those for (001) surfaces, with defect states lying in the middle of the band gap. The charge density of defect states shown in Fig. 5 indicates the formation of polarons near the surfaces. When in the surface layer (Fig. 5(a) and (c)) the dopants donate their additional electrons to the V ions in the subsurface layer, whereas the introduced polaron tends to be localized on Mo when Mo is positioned in the subsurface layer. This implies that the surface properties are generally the same for both (001) and (101) surfaces. Overall, the d orbital energy levels and the variation of electrostatic potential in the vicinity of surfaces are probably the main factors that dictate the localized positions of electron polarons generated by Mo/W dopants. Since the introduced polarons will not reside on W, we may anticipate that W dopants would be more effective than Mo in increasing the amount of charge carriers in BiVO_4_.

## Discussion

Through a detailed first principles analysis of electron polarons induced by Mo/W dopants in the bulk and near the surfaces of monoclinic BiVO_4_, a new insight is gained that polarons would be preferentially localized on V ions in the subsurface layer. This fact can be crucial to the understanding of the improved OER activity observed in previous experiments. The result of the experimental work^28^ has indicated that the photo-generated electrons and holes can be separated among different surfaces, and that electrons in the vicinity of the surfaces will be involved in the reduction of sacrificial acceptors (*e.g.*, Ag^+^) whereas holes take part in the OER. In this context, the transportation of both electrons and holes to the catalytic active sites can be of fundamental importance for the photocatalytic performance of BiVO_4_. Unlike the charge carriers in the bulk, the charge carriers migrating along the surfaces are more involved in the catalytic processes.^8^ Therefore, fast lanes for the migration of charge carriers near the surfaces can be the main feature that distinguishes the successful and unsuccessful BiVO_4_ photocatalysts. It is known that the conductivity of charge carriers is given by *σ* = c*μ, where *c* and *μ* are the concentration and mobility of the charge carriers.^18^ The electron polarons concentrated in the subsurface layer with Mo/W dopants near the surfaces can act as a reservoir for electron charge carriers, thus increasing the concentration and at last the conductivity of electrons along the surfaces. On the other hand, due to the critical role of the migration of holes in the surface layer where OER occurs, it would be more effective if there is less possibility for the holes to encounter electrons near the surfaces. The electrostatic potential of BiVO_4_ turns out to prevent electrons from localizing in the surface layer, thus retarding the electron–hole recombination process. These results can rationalize the observation that the superficial amount of dopants can exert more influence on photocatalytic performance than the dopant quantities in the bulk of BiVO_4_.

## Conclusion

In summary, we have calculated the electronic structures of Mo and W dopants in the bulk and near the (001) and (101) surfaces of BiVO_4_. According to our calculation results, small polarons would form when doping Mo/W in the bulk and near both surfaces. Through the DOS and charge density plots, we find that the additional electron introduced by both dopants in the vicinity of surfaces tends to be localized on the transition metal ion in the subsurface layer. The electron polaron would remain on Mo when Mo is doped beneath the surface layer, while W would denote the electron polaron to its nearby V ions. We attribute the above phenomena to the d-orbital energy levels of the transition metals and the variation of electrostatic potential near the surfaces of BiVO_4_. The localized electrons, *i.e.* polarons in BiVO_4_, in the subsurface layer can effectively improve the feasibility of electrons to the catalytic active sites on the surfaces, and mitigates the recombination between photo-generated electrons and holes, which can eventually lead to the promotion of photocatalytic performance of BiVO_4_.

## Conflicts of interest

There are no conflicts to declare.

## Supplementary Material

RA-009-C8RA09009B-s001
