# Supplementary material for: Electron polarons in the subsurface layer of Mo/W-doped BiVO4 surfaces
Source: RSC Adv. 2019 Jan 8;9(2):819–23. doi: 10.1039/c8ra09009b (PMC9059513; doi:10.1039/c8ra09009b)
Supplement: RA-009-C8RA09009B-s001 [file RA-009-C8RA09009B-s001.pdf]

Electronic Supplementary Material (ESI) for RSC Advances.
This journal is © The Royal Society of Chemistry 2018

Supporting Information for

Electron polarons in the subsurface layer of Mo/W-doped $BiVO_4$ surfaces

Jianhang Cen, Shunning Li*, Jiaxin Zheng, Feng Pan*

School of Advanced Materials, Peking University, Shenzhen Graduate School,

Shenzhen 518055, People's Republic of China

*Corresponding authors: S. Li (lisn@pkusz.edu.cn) and F. Pan

(panfeng@pkusz.edu.cn)

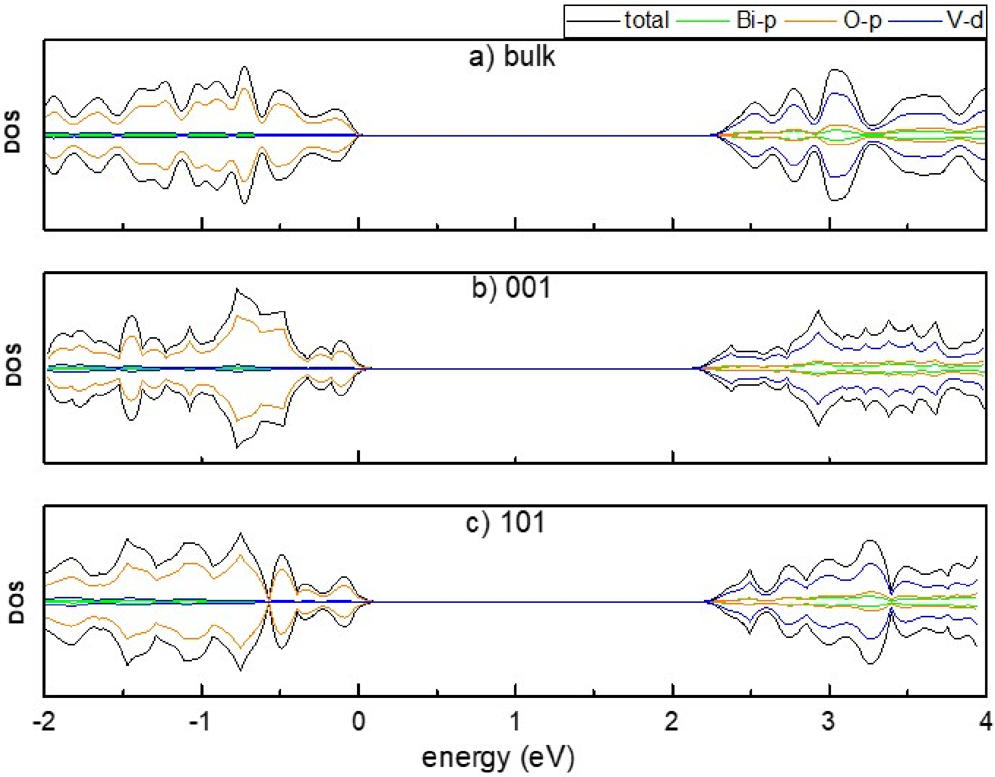

Fig. S1 DOS of (a) $BiVO_4$ bulk, (b) (001) surface, and (c) (101) surface.

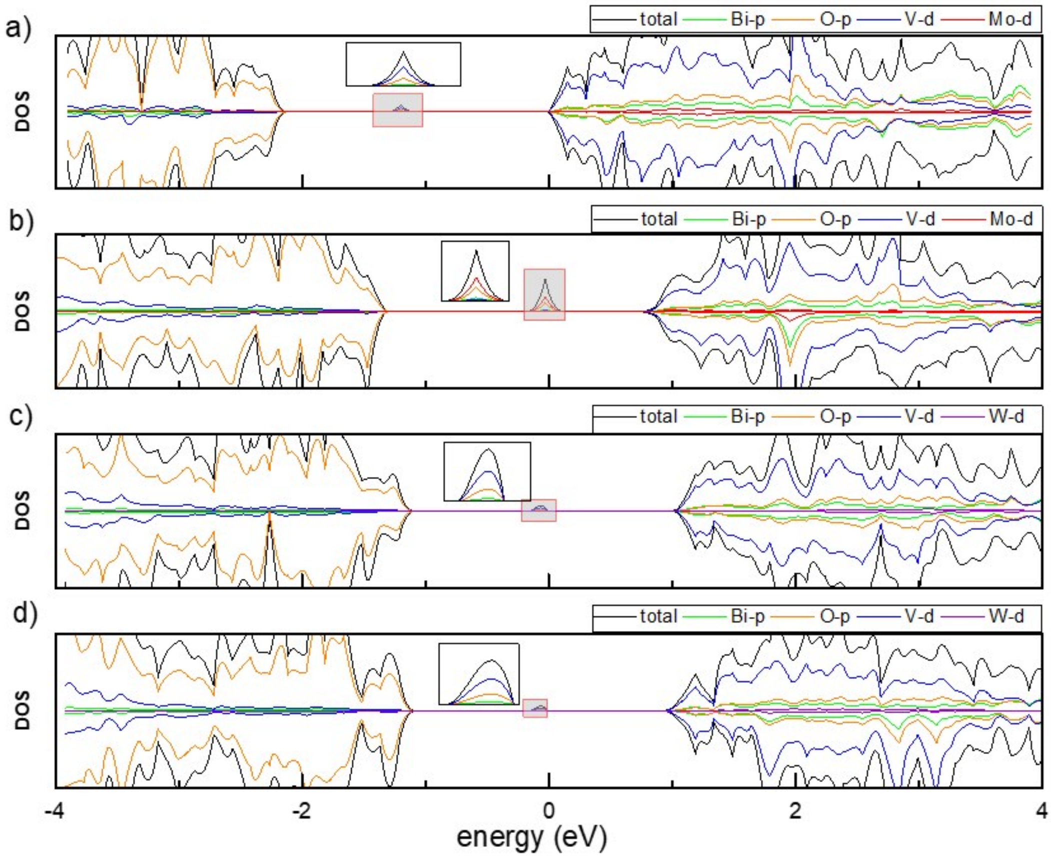

Fig S2 DOS for Mo/W-doped (001) surfaces. Mo doped in (a) the first and (b) second layers; W doped in (c) the first and (d) second layers.

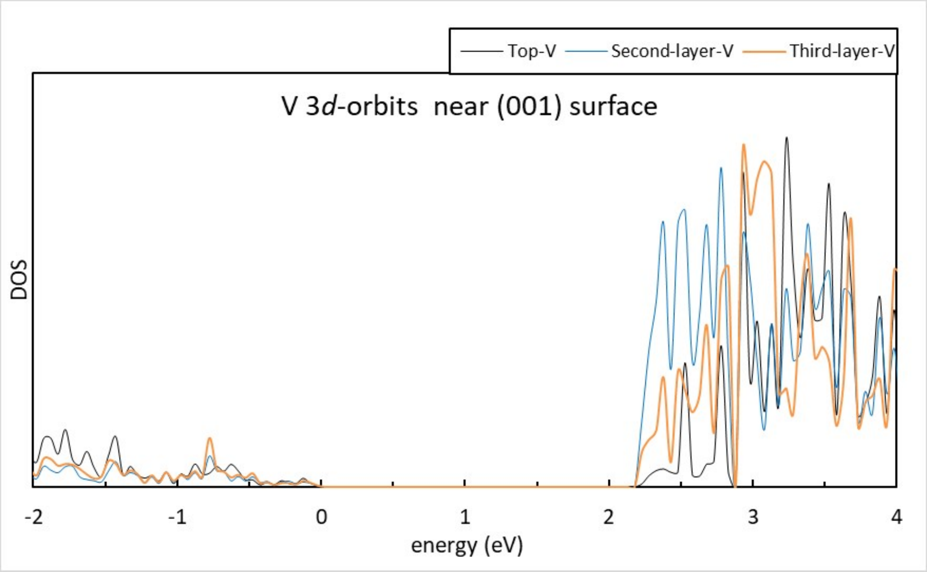

Fig. S3 Local DOS of V-*3d* orbitals near the (001) surface.

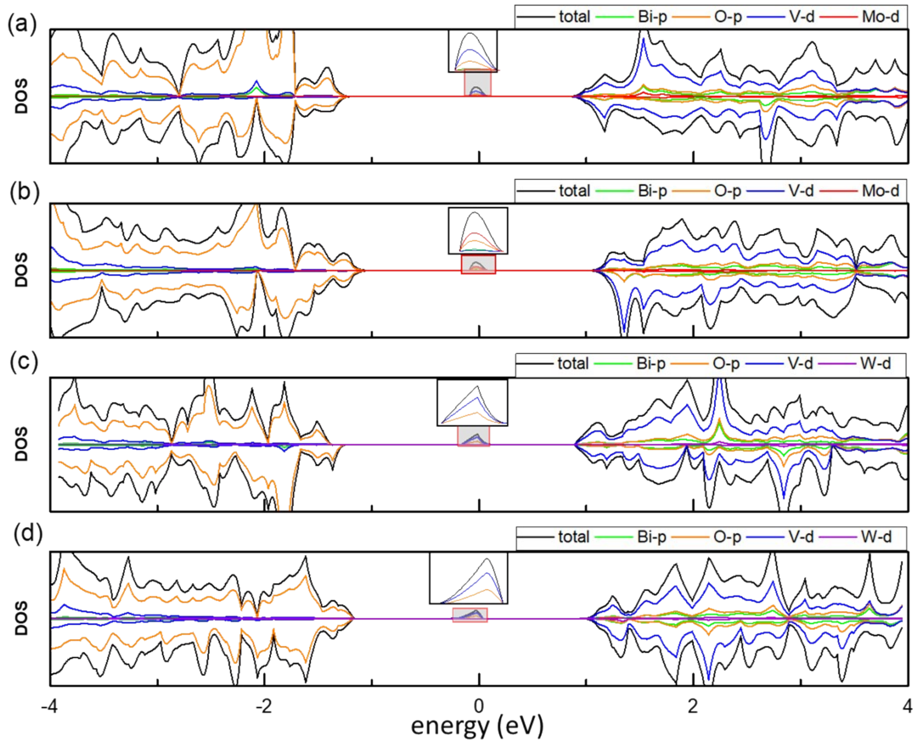

Fig S4 DOS for Mo/W-doped (101) surfaces. Mo doped in (a) the first and (b) second layers; W doped in (c) the first and (d) second layers.

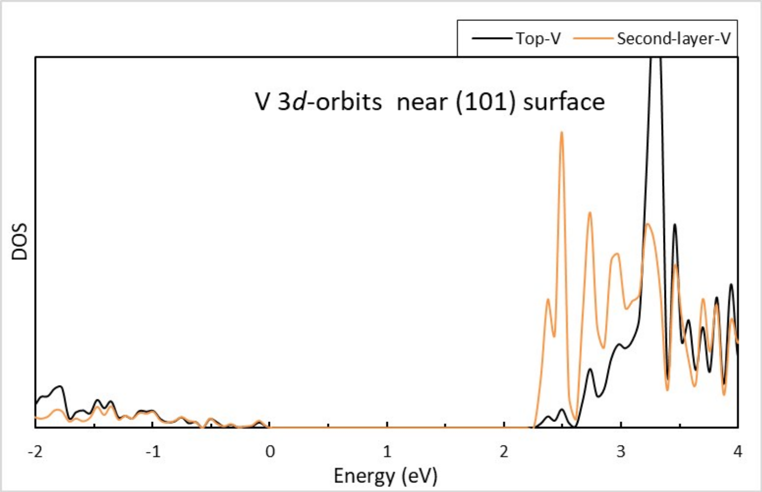

Fig. S5 Local DOS of V-*3d* orbitals near the (101) surface.
